# Experience with animals, religion, and social integration predict anthropomorphism across five countries

**DOI:** 10.1016/j.isci.2025.112693

**Published:** 2025-06-18

**Authors:** Federica Amici, Jose Luis Gomez-Melara, Bonaventura Majolo, Rufino Acosta-Naranjo, Patrícia Izar, Risma Illa Maulany, Putu Oka Ngakan, Shahrul Anuar Mohd Sah, Katja Liebal

**Affiliations:** 1 Human Biology & Primate Cognition Group, Institute of Biology, Leipzig University, Leipzig, Germany; 2 Department of Comparative Cultural Psychology, Max Planck Institute for Evolutionary Anthropology, Leipzig, Germany; 3 Department of Social Anthropology, University of Seville, Seville, Spain; 4 School of Psychology Sport Science and Wellbeing, University of Lincoln, Lincoln, UK; 5 Department of Experimental Psychology, University of São Paulo, São Paulo, Brazil; 6 Forestry Department, Hasanuddin University, Makassar, Sulawesi, Indonesia; 7 School of Biological Sciences, Universiti Sains Malaysia, Pulau Pinang, Malaysia

**Keywords:** Social sciences, Anthropology, Sociology

## Abstract

Conservation efforts are largely dependent on the amount of public concern for wildlife protection. One of the factors that best predict willingness to support conservation projects is anthropomorphism. Here, we administered questionnaires to a cross-cultural sample (*N* = 741), including adult participants from Brazil, Indonesia, Malaysia, Mexico, and Spain, to investigate the drivers of inter-individual variation in anthropomorphism. Anthropomorphism increased when participants had more “urban” experience with animals, lower social integration, higher allocentric tendencies, and lower formal education. Participants with higher exposure to monkeys were also less likely to attribute them the ability to feel pain, while considering them accountable for their actions. Religions emphasizing human similarity and inter-connection of all living beings predicted higher anthropomorphism, with the specific taxa and traits considered modulating these effects. Overall, this work contributes to better understanding the factors that explain variation in anthropomorphism and that might promote interest in other species and foster conservation efforts.

## Highlights

• Anthropomorphism toward animals increases with “urban” experience and social isolation
• Direct exposure to wildlife may trigger mechanisms to reduce cognitive dissonance
• Cultural traits like religion explain variation in anthropomorphism

## Graphical abstract

**Figure:**
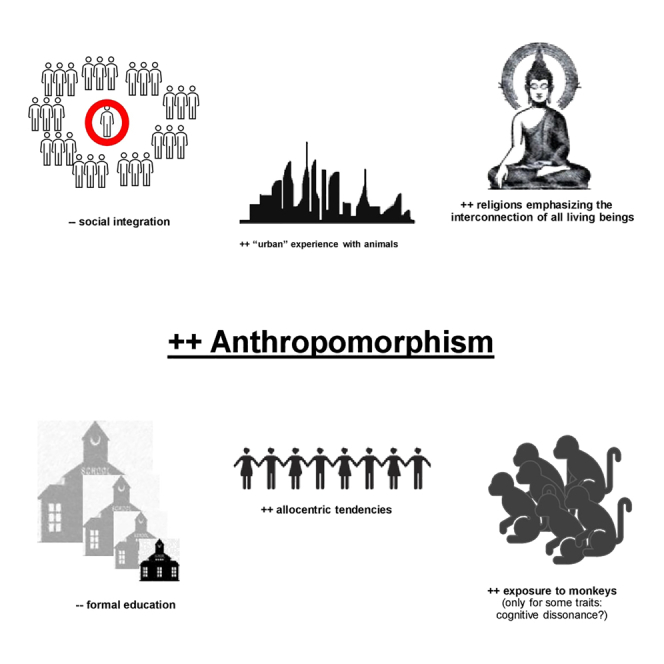

## Introduction

In the last decades, there has been a massive increase in anthropogenic pressure on nature.^1^^,^^2^^,^^3^ Changes in agricultural practices, urbanization, increasing population density, and resource consumption are significantly contributing to a reduction in the functional and structural complexity of ecosystems that are crucial for our health and survival.^4^^,^^5^^,^^6^ To date, several animal species across many taxa, from invertebrates to vertebrates, are considered vulnerable, endangered or critically endangered, with extinction rates corroborating the hypothesis of an ongoing mass extinction.^4^ Yet, current conservation efforts seem inadequate to stop this trend,^7^^,^^8^ and more action is urgently needed to avert the growing biodiversity loss and nature’s deterioration.^9^

Conservation efforts heavily rely on the knowledge and expertise of professionals working on the ground to safeguard animals and plants,^10^ but these efforts are also influenced by public interest and concern for biodiversity loss and wildlife protection. Public interest, in particular, depends on several factors. Humans, for instance, have a predisposition to prefer not only more “charismatic” (e.g., beautiful and impressive)^11^^,^^12^^,^^13^ species but also species that more closely resemble humans in terms of morphological, behavioral, and/or cognitive traits, and there is often greater support for conservation projects focusing on these species than on less charismatic or anthropomorphizable species.^14^^,^^15^^,^^16^^,^^17^^,^^18^^,^^19^ Anthropomorphism, in particular, can be defined as the tendency to attribute human traits to other living or non-living entities,^15^^,^^20^ and it is indeed known to enhance human interest and concern for other species.^16^^,^^21^ Anthropomorphism is a universal and partially automatic process, yet there are important differences across individuals in their tendency to anthropomorphize, which are likely shaped by social and cultural factors and by personal experiences.^20^^,^^21^^,^^22^^,^^23^

Although anthropomorphism can have positive implications for the conservation and welfare of other species, it can also lead to the misinterpretation of a species’ socio-ecology and behavior, leading to oversimplified assumptions about their characteristics and the wrong attribution of human-like traits, which can clearly lead to ineffective conservation strategies.^24^^,^^25^^,^^26^ Moreover, anthropomorphism can distract attention from species that are not similar to humans but have high conservation value, because their protection is crucial for maintaining biodiversity and supporting ecosystem functioning.^27^^,^^28^ Therefore, anthropomorphism can have both positive and negative effects on the conservation and welfare of other species, and understanding this process can help guide more effective and informed conservation strategies.

To date, little is known about the factors that explain inter-individual variation in anthropomorphic tendencies. The tendency to anthropomorphize, for instance, may vary depending on the knowledge we have about other species.^20^ Anthropomorphism is considered an inductive process of inference about non-human agents, in which knowledge about humans is used to explain properties of other species, when knowledge about these species is limited. However, as we acquire knowledge about other species, alternative knowledge structures become available, we become less likely to use knowledge about humans as a basis for induction, and anthropomorphism decreases.^20^^,^^21^^,^^29^ Knowledge on other species can clearly be acquired in many different ways, including formal or informal education, but also through direct experience with animals.^16^^,^^20^ Through education, individuals can gain a better understanding of a species’ true characteristics, grounded in biological and objective research, which can help reduce the tendency to wrongly attribute human-like traits to other species.^20^^,^^21^^,^^22^^,^^23^^,^^24^^,^^25^^,^^26^

The role of direct experience with animals on anthropomorphic tendencies, however, is debated. Traditionally, experience with animals has been considered to decrease anthropomorphic tendencies, because living in closer contact to nature would allow knowledge about other species to become more readily accessible when reasoning about animals.^20^ People from urban contexts (i.e., living in areas with higher population densities, infrastructure, and services, as compared to the surrounding rural areas)^30^ usually have lower contact and more limited knowledge of other species,^31^^,^^32^ so that they should be more likely than people from rural contexts to anthropomorphize when reasoning about other species.^20^^,^^33^^,^^34^ However, other authors highlight how urban contexts might facilitate the acquisition of a different kind of experience with animals and increase anthropomorphism.^16^ Modernization may be linked to social isolation and thus to the likelihood to recur to pet-keeping to fulfill the need of social connection.^16^^,^^35^ Such an increase in pet-keeping over the last decades^36^ may have provided humans with opportunities to form close affiliative bonds with other species, increasing the perception of their similarity to humans.^16^ In line with this view, several studies have shown a link between pet-keeping and empathy toward other species.^37^^,^^38^^,^^39^^,^^40^ In urban contexts, therefore, people might have reduced direct interactions with wildlife, with experiences primarily occurring through media, recreational activities, or encounters with animals that have adapted to urban settings. This shift to more “urban” experience with animals (i.e., during safe interactions with captive, domesticated and/or virtual animals, like in zoos and aquariums, with pets, or on internet) might foster anthropomorphic tendencies, by promoting affiliative bonds with animals and increasing perception of similarity to other species.^16^ In contrast, direct exposure to other species, which is more likely in more rural contexts, might decrease anthropomorphism, by providing alternative knowledge structures to rely on.^20^

The tendency to anthropomorphize may vary depending on the socio-cultural context. Different cultures have specific values, norms, or beliefs about the attributes of other species, which influence exposure to and knowledge about animals.^16^^,^^20^^,^^29^^,^^41^ Cultural values, norms, and beliefs that highlight the role of other animals as human commodities, for example, may hinder human perception of similarity to them and reduce anthropomorphism.^16^ In contrast, cultural settings that emphasize human similarity to other species and the inter-connection of all living beings, foster the attribution of human traits to other animals.^16^^,^^42^ Religions, in particular, can provide an extensive set of values, norms, and beliefs that orient our relationship with animals.^43^ Monotheistic religions often adopt worldviews that place humans at the center and view animals as secondary creations primarily meant to serve human interests and needs. Conversely, non-monotheistic religions and other belief systems place less emphasis on the distinction between humans and other species^44^ and may therefore be expected to promote the attribution of human traits to other species.

Social integration also affects anthropomorphism.^20^^,^^21^ Individuals who are not well integrated in their group and who experience loneliness and social isolation partially fulfill their need of social connection by attributing human traits to animals and other non-human agents.^20^^,^^45^^,^^46^ For instance, chronically lonely individuals are more likely to anthropomorphize their pets as compared to socially integrated individuals.^47^ Moreover, the relationship between social integration and anthropomorphism may be mediated by an individual’s level of self-reliance or interdependence. In particular, while anthropomorphism tends to increase with social isolation, this increase may be more pronounced in allocentric individuals, who are more interdependent and less self-reliant than idiocentric individuals, and who might experience greater stress from social isolation.^20^^,^^21^^,^^48^^,^^49^^,^^50^^,^^51^ Anthropomorphic tendencies also vary depending on the specific non-human agents they are attributed to.^20^ Anthropomorphism is thought to be stronger toward species that are more “charismatic,”^11^^,^^12^^,^^13^^,^^52^ that are perceived as being more similar to humans in their morphology or behavior,^53^ and/or that are phylogenetically closer to humans,^14^^,^^54^ like non-human primates (hereafter primates). Humans attribute higher cognitive skills to taxa that are morphologically more similar to us^55^ and are more willing to invest in the conservation of phylogenetically closer species to humans.^17^^,^^18^^,^^56^ Therefore, it is likely that individual anthropomorphic tendencies are mediated by the specific species they refer to, including their morphological and behavioral similarity to humans.

Finally, anthropomorphism encompasses a variety of different traits that can be attributed to other species, including morphological, behavioral, and cognitive traits.^19^^,^^20^^,^^21^ Although most authors restrain anthropomorphism to the attribution of mental states (e.g., emotions, intentions, consciousness),^20^^,^^21^ there is still a lot of variation across these traits, and it is likely that there are differences also within individuals in the tendency to attribute them. For instance, whereas secondary emotions (e.g., shame and hope) are usually perceived as being uniquely human, primary emotions (e.g., fear and anger) are more often attributed also to other species.^21^^,^^57^ Therefore, it is possible that individual anthropomorphic tendencies are modulated by the specific traits considered.

In this study, we used a cross-cultural sample (*N* = 741), including adult participants from Brazil, Indonesia, Malaysia, Mexico, and Spain (Table 1), to investigate the drivers of inter-individual variation in anthropomorphic tendencies. We selected these countries because they allowed us to cover three continents, ensured enough variation in the variables we wanted to test, and provided the co-authors with the chance to recruit participants through their habitual channels (see STAR Methods). Based on existing literature, we made the following hypotheses and predictions. First, we hypothesized that the tendency to anthropomorphize varies depending on the knowledge humans have of other species. In particular, we predicted that anthropomorphic tendencies would be higher in individuals with lower formal education (Prediction 1a) and lower exposure to animals (Prediction 1b), as they might have more limited knowledge of other species. However, we predicted anthropomorphic tendencies to be higher also in individuals with more extensive “urban” experience with animals (Prediction 1c), as this could promote affiliative bonds with animals and increase anthropomorphism. Second, we hypothesized that the tendency to anthropomorphize varies depending on the socio-cultural context experienced. In particular, we predicted that anthropomorphic tendencies would be lower in individuals who follow monotheistic religions that generally describe animals as being subordinate to human interests and needs (i.e., Christianity and Islam; Prediction 2). Third, we hypothesized that the tendency to anthropomorphize varies depending on an individual’s social integration. In particular, we predicted that anthropomorphism would be higher in individuals with lower social integration (Prediction 3a), who experience higher social isolation, and in more allocentric individuals (Prediction 3b), who may more strongly respond to periods of social isolation. Finally, we hypothesized that anthropomorphic tendencies are modulated by the specific species and traits considered. In particular, we predicted that the tendency to anthropomorphize would be stronger toward phylogenetically closer species (Prediction 4a) and for traits that are usually considered not to be uniquely human (e.g., primary as compared to secondary emotions; Prediction 4b).

**Table 1:** Study participants

Country	Brazil	Indonesia	Malaysia	Mexico	Spain	TOTAL
Participants	156	248	118	111	108	741
Females:males	79:44	146:101	59:18	60:26	50:51	394:240
Age (years)	43.9 ± 15.4	23.9 ± 9.1	22.2 ± 2.8	35.6 ± 10.8	21.6 ± 9.9	28.8 ± 13.6
Religion:						
Atheists/agnostics	74	0	5	43	54	176
Buddhists/Hindus	2	1	16	1	1	21
Christians	24	36	4	33	40	137
Muslims	0	211	49	0	0	260
Other religion	17	0	1	5	2	25
Formal education:						
No primary school	0	1	0	0	0	1
Primary school	0	15	0	0	0	15
Secondary school	39	218	33	14	88	392
Bachelor degree	13	14	41	34	7	109
Higher degrees	69	0	1	38	2	110
Social integration:						
Allo-/idiocentrism	0.5 ± 0.1	0.6 ± 0.1	0.6 ± 0.1	0.5 ± 0.1	0.5 ± 0.1	0.5 ± 0.1
Social integration	1.2 ± 0.5	2.1 ± 0.8	1.3 ± 0.5	1.4 ± 0.6	1.5 ± 0.6	1.6 ± 0.8

Demographic characteristics of the study participants: sample size (N) or, when applicable, average and standard deviation (SD).

## Results

### Attribution of free will to monkeys and other animals

Participants attributed on average (±SD) a high level of free will, assigning a score of 0.73 ± 0.21 to both monkeys and other animals. In Model 1, the full model significantly differed from the null model **(**GLMM, χ^2^ = 48.55, *df* = 19, *p* < 0.001). Participants were more likely to attribute free will to animals (including monkeys) when they were more allocentric than idiocentric (*p* = 0.004) and when they had a lower level of formal education (*p* = 0.037) and more “urban” experience with animals (*p* < 0.001; Table S2).

### Attribution of intentions to monkeys and other animals

Participants attributed on average a high level of intentionality, assigning a score of 0.75 ± 0.19 to monkeys and of 0.74 ± 0.20 to other animals. In Model 2, we found a significant difference between the full and the null models **(**GLMM, χ^2^ = 47.99, *df* = 19, *p* < 0.001). Participants were more likely to attribute intentions to monkeys than to other animals (*p* = 0.027), and they were also overall more likely to attribute intentions to animals (including monkeys) if having more “urban” experience with animals (*p* = 0.008; Table S2).

### Attribution of consciousness and mind to monkeys and other animals

**Figure 1:**
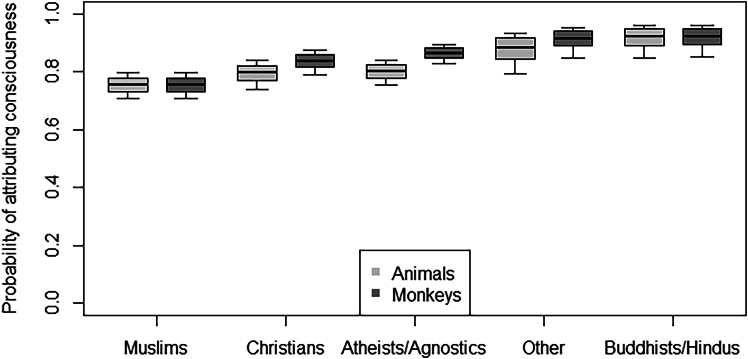
Attribution of consciousness to monkeys and other animals Probability of attributing consciousness to animals (light gray bars) and specifically to monkeys (dark gray bars), as a function of participants’ religion. The thick lines of the boxplots represent the mean probabilities as estimated by models 3 and 4. The ends of the boxes represent the estimated standard errors, and the ends of the whiskers represent the 95% confidence intervals. Only atheists/agnostics and Christians were more likely to attribute consciousness to monkeys than to other animals.

**Figure 2:**
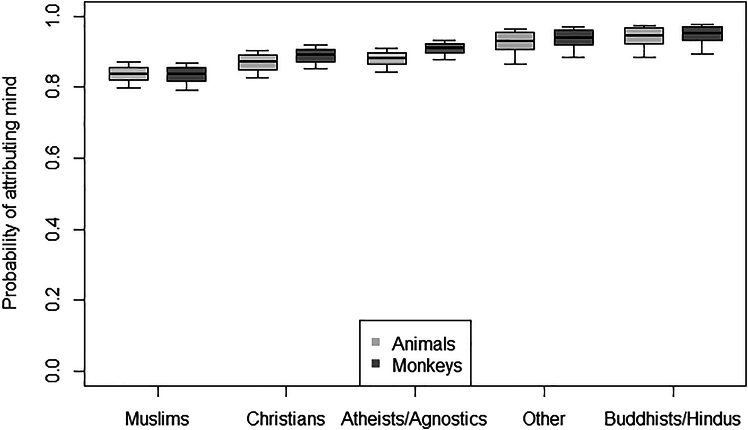
Attribution of mind to monkeys and other animals Probability of attributing mind to animals (light gray bars), specifically to monkeys (dark gray bars), as a function of participants’ religion. The thick lines of the boxplots represent the mean probabilities as estimated by models 3 and 4. The ends of the boxes represent the estimated standard errors, and the ends of the whiskers represent the 95% confidence intervals. Only atheists/agnostics and Christians were more likely to attribute mind to monkeys than to other animals.

Participants attributed on average a high level of consciousness and theory of mind, assigning a consciousness score of 0.76 ± 0.20 to monkeys and of 0.73 ± 0.20 to other animals, and a mind score of 0.80 ± 0.17 to monkeys and of 0.79 ± 0.18 to other animals. In Models 3 and 4, the full models significantly differed from the corresponding null models (Model 3: GLMM, χ^2^ = 68.10; Model 4: GLMM, χ^2^ = 48.33; both *df* = 19, *p* < 0.001). Participants were overall more likely to attribute both consciousness and mind to animals (including monkeys) when they had more “urban” experience with animals (Model 3: *p* = 0.007; Model 4: *p* = 0.002; Table S2). Moreover, religion modulated variation in the probability of attributing consciousness and mind to monkeys rather than other animals (Model 3: *p* < 0.001; Model 4: *p* = 0.002; Table S2). In particular, the probability of attributing consciousness and mind to animals (including monkeys) was overall highest for Buddhists/Hindus and participants with other religions, followed by atheists/agnostics and Christians, and finally by Muslims. Post-hoc tests showed that only atheists/agnostics (Model 3: *p* < 0.001; Model 4: *p* < 0.001) and Christians (Model 3: *p* = 0.003; Model 4: *p* = 0.013) were more likely to attribute consciousness (Figure 1) and mind (Figure 2) to monkeys than to other animals, in contrast to other participants who showed no significant difference between taxa (Table S3).

### Attribution of emotions to monkeys and other animals

Participants attributed on average a high level of emotion, assigning a score of 0.87 ± 0.14 to monkeys and of 0.86 ± 0.14 to other animals. In Model 5, we found a significant difference between the full and the null models **(**GLMM, χ^2^ = 41.29, *df* = 19, *p* = 0.002). Participants were more likely to attribute emotions to monkeys than to other animals (*p* = 0.038), and they were also overall more likely to attribute emotions to animals (including monkeys) if having more “urban” experience with animals (*p* < 0.001) and if experiencing lower social integration (*p* = 0.050; Table S2).

### Attribution of other anthropomorphic traits to monkeys

On average, participants attributed to monkeys a score of 0.90 ± 0.11 for their ability to experience pain, 0.84 ± 0.16 for their ability to experience primary emotions, 0.78 ± 0.17 for their ability to understand others’ intentions, 0.77 ± 0.20 for their physical similarity to humans, 0.74 ± 0.20 for their ability to deceive others, 0.73 ± 0.19 for their ability to understand and share others’ feelings, 0.66 ± 0.23 for their ability to experience secondary emotions, and 0.60 ± 0.21 for their ability to distinguish good and evil. In Model 6, the full model significantly differed from the null model **(**GLMM, χ^2^ = 1251.30, *df* = 79, *p* < 0.001). In particular, the interactions between anthropomorphic trait and participant’s religion (*p* < 0.001), exposure to monkeys (*p* < 0.001), experience with animals (*p* = 0.034), and social integration (*p* = 0.015) were all significant (Table S2).

**Figure 3:**
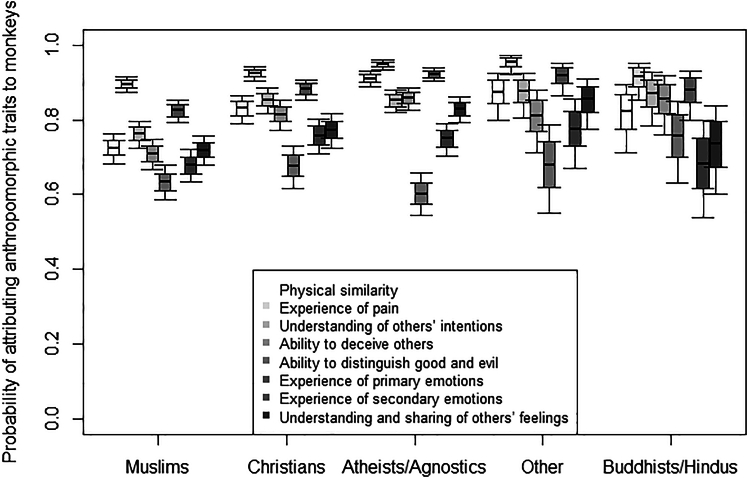
Attribution of anthropomorphic traits to monkeys Probability of attributing anthropomorphic traits to monkeys as a function of participants’ religion, separately for each trait (i.e., physical similarity, experience of pain, understanding of others’ intentions, ability to deceive others, ability to distinguish good and evil, experience of primary emotions, experience of secondary emotions, understanding and sharing of others’ feelings). The thick lines of the boxplots represent the mean probabilities as estimated by model 6. The ends of the boxes represent the estimated standard errors, and the ends of the whiskers represent the 95% confidence intervals. Muslims were less likely to attribute the ability to deceive others and understand others’ intentions to monkeys than atheists/agnostics and Christians; Muslims were less likely to attribute primary emotions, other feelings, and pain to monkeys than atheists/agnostics and participants with other religions; physical similarity was more likely attributed by atheists/agnostics than by Christians and Muslims and less likely by Muslims than Christians and participants with other religions.

First, Muslims were less likely to attribute the ability to deceive others and to understand others’ intentions to monkeys, as compared to atheists/agnostics (ability to deceive others: *p* < 0.001; understanding of others’ intentions: *p* = 0.009) and Christians (ability to deceive others: *p* = 0.008; understanding of others’ intentions: *p* = 0.006; Table S3; Figure 3). Moreover, Muslims were less likely to attribute primary emotions, other feelings, and pain to monkeys, as compared to atheists/agnostics (primary emotions: *p* < 0.001; other feelings: *p* = 0.002; pain: *p* < 0.001) and participants with other religions (primary emotions: *p* = 0.037; other feelings: *p* = 0.044; pain: *p* = 0.045; Table S3; Figure 3). Furthermore, physical similarity was more likely attributed by atheists/agnostics than by Christians and Muslims (both *p* < 0.001) and less likely by Muslims than Christians (*p* = 0.005) and participants with other religions (*p* = 0.014; Table S3; Figure 3).

**Figure 4:**
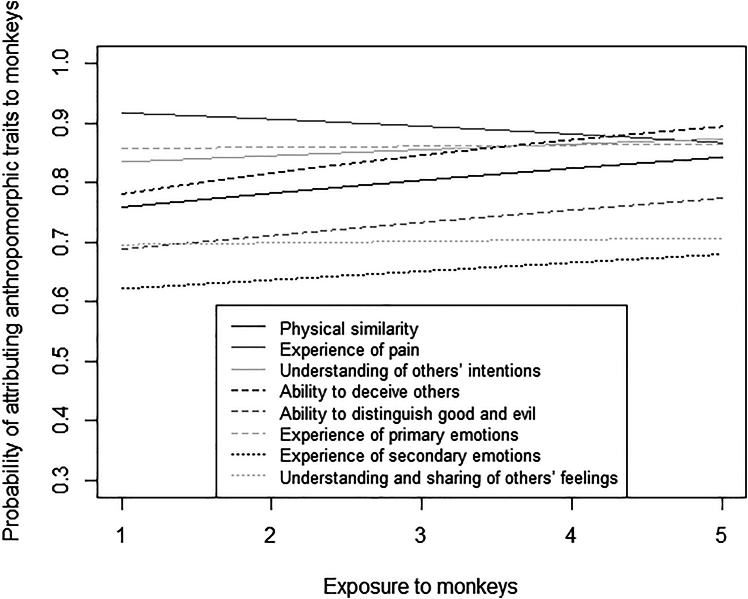
Attribution of anthropomorphic traits to monkeys Probability of attributing anthropomorphic traits to monkeys as a function of participants’ exposure to them, separately for each trait (i.e., physical similarity, experience of pain, understanding of others’ intentions, ability to deceive others, ability to distinguish good and evil, experience of primary emotions, experience of secondary emotions, understanding and sharing of others’ feelings). The lines represent model 6 for the different traits. Participants with higher exposure to monkeys were more likely than participants with lower exposure to monkeys to attribute them physical similarity, ability to deceive others, and ability to distinguish good and evil but less likely to attribute them pain.

**Figure 5:**
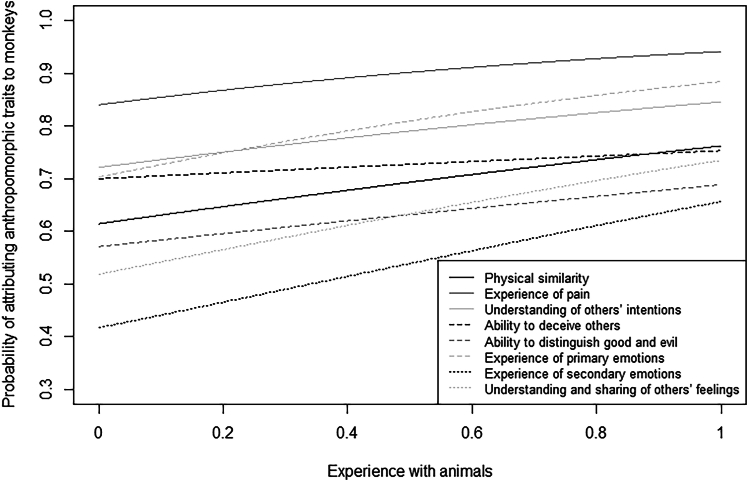
Attribution of anthropomorphic traits to monkeys Probability of attributing anthropomorphic traits to monkeys as a function of participants’ experience with animals, separately for each trait (i.e., physical similarity, experience of pain, understanding of others’ intentions, ability to deceive others, ability to distinguish good and evil, experience of primary emotions, experience of secondary emotions, understanding and sharing of others’ feelings). The lines represent model 6 for the different traits. The probability of attributing physical similarity, pain, primary emotions, secondary emotions, understanding of others’ intentions, and understanding and sharing of others’ feelings to monkeys was higher for participants having more rather than less “urban” experience with animals.

**Figure 6:**
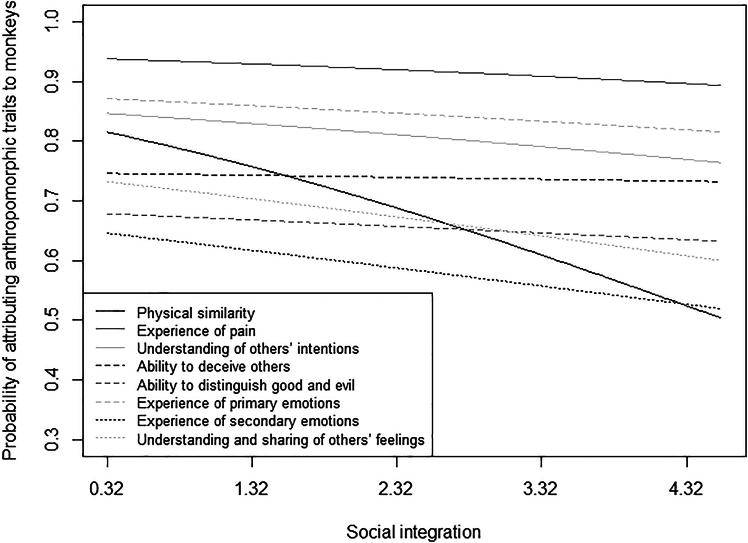
Attribution of anthropomorphic traits to monkeys Probability of attributing anthropomorphic traits to monkeys as a function of participants’ experience with animals, separately for each trait (i.e., physical similarity, experience of pain, understanding of others’ intentions, ability to deceive others, ability to distinguish good and evil, experience of primary emotions, experience of secondary emotions, understanding and sharing of others’ feelings). The lines represent model 6 for the different traits. The probability of attributing physical similarity to monkeys was higher for individuals experiencing lower than higher social integration.

Participants with higher exposure to monkeys were more likely than participants with lower exposure to monkeys to attribute them physical similarity (*p* = 0.005), ability to deceive others (*p* < 0.001), and ability to distinguish good and evil (*p* = 0.015), but less likely to attribute them pain (*p* = 0.005; Figure 4). The probability of attributing physical similarity (*p* = 0.010), pain, primary emotions, secondary emotions (all *p* < 0.001), understanding of others’ intentions (*p* = 0.006), understanding and sharing of others’ feelings (*p* < 0.001) to monkeys was higher for participants having more rather than less “urban” experience with animals (Figure 5). Finally, the probability of attributing anthropomorphic traits to monkeys was generally higher for individuals experiencing lower than higher social integration, but significantly so only for physical similarity (*p* < 0.001; Figure 6).

## Discussion

In this study, we found evidence that anthropomorphic tendencies varied across adult participants in five different countries based on their knowledge of other species, on the socio-cultural context they experienced, on their social integration, and on the specific species and traits considered. In line with our first hypothesis, the tendency to anthropomorphize animals depended on participants’ knowledge: participants with lower formal education, in particular, were more likely than people with higher formal education to attribute free will to animals (Prediction 1a). These findings are in line with previous work suggesting that anthropomorphism increases in conditions of causal uncertainty and/or high cognitive load, when knowledge about other species may be limited, and people might more heavily recur to human traits to explain properties of other species.^20^^,^^21^^,^^29^

In contrast, the effect of exposure to animals on participants’ anthropomorphism was more complex than we had predicted. In particular, participants who were more strongly exposed to monkeys had not only a lower probability to attribute them sensitivity to pain but also a higher probability to attribute them physical similarity, ability to deceive others, and ability to distinguish good and evil (largely in contrast to Prediction 1b). In contrast to our hypothesis, therefore, direct exposure to monkeys did not simply increase human knowledge about animals, reducing anthropomorphic tendencies; rather, direct exposure to monkeys appeared to trigger the attribution of specific human traits and the denial of others (i.e., sensitivity to pain). Participants with higher exposure to monkeys were more likely to perceive them as being deceptive and morally responsible for their actions (i.e., able to distinguish good and evil)^57^^,^^58^ but also less likely to feel pain. Although the spectrum of human-animal interactions is large and multifaceted, and includes positive interactions (e.g., appreciation, reverence), these interactions can also be conflictual, for instance when animals pose or are perceived to pose a threat to human health, food, or property.^58^^,^^59^^,^^60^^,^^61^^,^^62^^,^^63^ In these cases, humans might consider other species responsible for their actions and still maintain moral distance by denying them sensibility to pain.^14^^,^^64^^,^^65^^,^^66^^,^^67^^,^^68^^,^^69^ In Western communities, these mechanisms are thought to reduce the uncomfortable feeling of considering the harm that humans might infer to animals during conflictual events (e.g., meat consumption).^66^ In this study, these mechanisms might have been used by participants who were more likely to have (or have had) negative interactions with monkeys, for instance in the form of crop raiding or other threats to health or properties.^58^^,^^59^^,^^60^^,^^61^^,^^62^^,^^63^

As predicted, individuals who had more extensive “urban” experience with animals also had higher anthropomorphic tendencies (Prediction 1c). “Urban” experience with animals (e.g., having pets, petting them, visiting zoos), in particular, increased the probability that participants would attribute free will, intentions, consciousness, mind, and emotions to animals and also that they would attribute physical similarity, pain, primary and secondary emotions, understanding of others’ intentions, and understanding and sharing of others’ feelings to monkeys. These results strongly support the hypothesis that “urban” experience with animals may provide humans with more controlled, safe, and non-conflictual opportunities to interact with animals, which may promote affiliative bonds with other species and increase the perception of their similarity to humans and the tendency to attribute them human traits.^16^ Future studies should ideally better disentangle which aspects of “urban” experience are more clearly linked to anthropomorphic tendencies. In our study, “urban” experience included several aspects of human-animal interactions that may occur in “urban” contexts, during safe interactions with captive, domesticated, and/or virtual animals. Our questions, in particular, measured several aspects of “urban” experience, like pet ownership (i.e., having pets, petting them, and considering them as friends), virtual encounters with animals in the media (i.e., knowledge of movies and books with animal characters and exposure to news about animals on TV or in internet), and exposure to animals in other “urban” contexts (i.e., visiting zoos and learning about animals at school). Several studies have shown that anthropomorphism is affected by the extent to which people encounter media featuring anthropomorphized animals.^70^^,^^71^^,^^72^ While our results are in line with these findings, future work should better assess the exact role of all these different “urban” experiences on anthropomorphism.

In line with our second hypothesis, the tendency to anthropomorphize animals varied depending on the socio-cultural context that our study participants experienced and, in particular, on their religion. The probability of attributing consciousness and mind to animals, for instance, was lowest in Muslims, intermediate in Christians and atheists/agnostics, and highest in Buddhists/Hindus and participants with other religions. Similarly, Muslims were less likely to attribute some human traits to monkeys, as compared to Christians and atheists/agnostics (e.g., ability to deceive others and understanding of others’ intentions) and as compared to atheists/agnostics and participants with other religions (primary emotions, other feelings, and pain). These results should be interpreted with extreme caution, because the limited size of our study sample forced us to group participants into very broad categories (e.g., “Muslims”, “Buddhists/Hindus”), failing to properly reflect the inherent pluralism and diversity of the religions considered. Despite these important limitations, our results seem to support the prediction (Prediction 2) that anthropomorphic tendencies decrease in individuals with monotheistic religions, which may tend to describe animals as subordinate to humans, partially hindering human perception of similarity.^16^ In contrast, religions that emphasize human similarity to other species and the inter-connection of all living beings might foster the attribution of human traits to other animals.^16^^,^^42^^,^^44^

In line with our third hypothesis, we found that anthropomorphic tendencies in our study varied depending on participants’ social integration. In particular, participants experiencing lower social integration were more likely to attribute human traits (e.g., emotions and physical similarity) to monkeys and other animals (Prediction 3a), whereas more allocentric individuals were more likely than idiocentric ones to attribute free will to animals (Prediction 3b). These results highlight variation in the allocation of human traits to animals, depending on the traits considered, and may further suggest that, across countries, anthropomorphism is more likely not only in individuals who experience higher social isolation and may fulfill their need of social belonging by bonding with other species^16^^,^^20^^,^^29^^,^^45^^,^^47^ but also in more allocentric individuals, who being more interdependent might more strongly respond to periods of social isolation.^20^^,^^29^

Finally, our study showed that anthropomorphic tendencies were modulated by the specific species and traits considered. In particular, participants were more likely to attribute intentions and emotions (and partially consciousness and mind) to monkeys, as compared to other animals (in line with Prediction 4a). Monkeys are not only phylogenetically close to humans but also share several morphological and behavioral attributes with them. These results confirm that phylogenetic closedness and morphological and/or behavioral similarity can increase human anthropomorphic tendencies.^14^^,^^53^^,^^55^^,^^56^^,^^73^^,^^74^^,^^75^ In the future, it would be interesting to include a higher number of species from different taxa, not only to explore the relative contribution of phylogenetic distance and morphological and behavioral similarity to anthropomorphic tendencies but also to test whether these patterns are consistent across countries and cultural settings. Moreover, our results evidenced significant variation in the traits that participants attributed to animals (in line with Prediction 4b), with knowledge of other species, socio-cultural context, and social integration often predicting the attribution of only some specific traits. These findings call for more caution when assessing anthropomorphic tendencies, because individual responses might strongly vary based on the specific traits and species considered.

### Limitations of the study

Overall, our study identified several possible drivers of inter-individual variation in anthropomorphic tendencies, which appear to play a significant role across different countries. Yet, more research is needed to confirm our findings. In particular, future studies should increase sample size to allow finer-grained analyses of possible variables predicting anthropomorphic tendencies (e.g., religious and cultural beliefs about animals and types of previous experience with animals) and the inclusion of more countries and cultures. Moreover, future research should better disentangle to what extent the attribution of human traits to other species really reflects anthropomorphic interpretations rather than objective understanding of their skills and behaviors.^23^ Future studies should also explore more thoroughly what participants specifically mean when attributing certain human traits to other animals. For example, terms like “consciousness,” which are commonly used in research on anthropomorphism,^21^ can be interpreted in various ways, as they are broad and multifaceted concepts. Finally, one of the nine questions we used to assess social integration measured the number of contacts participants had in Facebook. Although this approach is widely used in research, also with young participants,^76^^,^^77^ future studies might include other social media to better account for inter-individual variation in the specific kind of social media that participants use. Despite the limitations of our study, our work contributes to better understanding the factors that explain variation in anthropomorphism across countries. This has important implications for nature protection initiatives, because anthropomorphism plays a crucial role on the extent to which people are interested in other species and it thus affects conservation efforts.^17^^,^^18^^,^^19^^,^^56^^,^^78^ The attribution of human traits to nature, for instance, is linked to an increase in human connection to nature and also in the willingness to engage in conservation behavior.^79^ Moreover, anthropomorphism can be used to increase humans’ moral valuation of other animals,^80^ foster the attribution of rights to these species,^29^ and ultimately promote their welfare.^81^ There is growing evidence that conservation initiatives need to be tailored on the specific culture targeted by the initiative, in order to be effective.^82^^,^^83^^,^^84^ Our study contributes to this perspective by suggesting that, within and across cultures, individual differences in anthropomorphism may influence the success of conservation initiatives. Therefore, these differences should be considered when designing conservation policies and education programs, to ensure that public interest aligns with, and actively supports conservation priorities established through rigorous scientific research. Gaining insight on the drivers of anthropomorphism, therefore, might provide a power tool to more effectively implement initiatives in favor of animal welfare and nature protection that are fine-tuned on the specific community group and socio-cultural context of the humans involved.

## Resource availability

### Lead contact

Further information and requests for resources and reagents should be directed to and will be fulfilled by the lead contact, Federica Amici (amici@eva.mpg.de).

### Materials availability

This study did not generate new unique reagents.

### Data and code availability

• Data are provided as supplemental information.
• Code is provided as supplemental information.
• No other resources have been used for this work.

## Acknowledgments

We are grateful to Laura Amores, Iván Periañez, Jose María Sánchez, Virginia Alba, Jose Luis Escacena, and Plácido Fernandez-Viagas for their help during data collection. We thank the whole team at CACTUS-lab for their help and generosity adapting our questionnaire to the online version of Ethnoap. We are also grateful to the 10.13039/100014440Ministerio de Ciencia, Innovación y Universidades for funding Jose Gómez-Melara (FPU 16/02878) during his PhD and to the 10.13039/501100001807FAPESP (14/13237-1) for funds allowing Patrícia Izar to access local communities during this research.

## Author contributions

F.A., J.L.G.-M., B.M., and K.L. conceptualized the paper; J.L.G.M., P.I., and S.A.M.S. collected the data, with the help from R.A.-N., R.I.M., and P.O.N. F.A. ran the analyses and wrote the paper, with extensive feedback from all the other co-authors, who agree on the content and interpretation of the results.

## Declaration of interests

The authors declare no competing interests.

## STAR★Methods

### Key resources table

**Table:** 

REAGENT or RESOURCE	SOURCE	IDENTIFIER
**Software and algorithms**		

R Software	Team, R.C.^85^	https://www.r-project.org/
EthnoApp	EthnoApp^86^	https://ethnoapp.com

### Experimental model and study participant details

We collected data in five countries (i.e. Brazil: N=156, Indonesia: N=248, Malaysia: N=118, Mexico: N=111, and Spain: N=108). The total sample size was therefore N=741, and as all participants were provided with the same questionnaire, there was no allocation to specific experimental groups. We started the study using a printed version of the questionnaire in Indonesia and partially in Spain. However, due to the outbreak of the COVID-19 pandemic, we had to stop direct interactions with participants and instead use an online version of the questionnaire in the software Ethnoap. In Indonesia, we opportunistically recruited participants in the Bira Bonto Bahari beach road area (N = 100), and at the Hasanuddin University campus (N = 145). In Spain, we first recruited participants at the University of Seville campus, but as the COVID-19 pandemics started, we recruited participants online, by distributing the Ethnoap link through the University of Seville online-class platform. In Malaysia and Brazil, we recruited participants through social media, by advertising the questionnaire to acquaintances in both urban and rural communities.

To ensure that the different way of recruiting participants did not bias our results, we compared the responses given by participants who were recruited in person versus those who were recruited online in Spain (i.e., the only country in which we tested participants both online and in person). The comparison revealed no difference between the two groups in participants’ anthropomorphism, exposure to and experience with animals, social integration and allocentric/idiocentric tendencies (see Table S1 in supplemental information for all the means, standard deviations and results of the Mann Whitney tests). Thus, we included all participants in the following analyses, regardless of the modality we used to recruit them. Sample size was deemed appropriate based on sample sizes used in the literature on anthropomorphism. Although we aimed to recruit a sample representative of the general population, our final sample was biased towards younger individuals with higher formal education. In Table 1, we report more details about the demographic distribution of the participants in the five countries, including their gender, age, religion and education.

### Method details

All procedures were performed in compliance with relevant national laws and institutional guidelines, and were approved by the Research Ethics Committee of the University of Sevilla (CEIUS), Spain. Our study adhered to the guidelines set forth in the Code of Good Research Practices, as approved by the Doctoral Commission of the Vice-Rectorate for Research. Further ethical approval was provided by the Kementarian Negara Riset dan Teknologi Republik Indonesia (RISTEK). In all communities, participation was voluntary and completely anonymous. Informed consent was obtained from all subjects before testing started. Participants were informed about the purpose of the study and were able to withdraw their participation at any time.

All participants received a questionnaire in their native language. The questionnaire was first prepared in English, and then translated into Portuguese, Indonesian, Malaysian and Spanish by a native speaker of the relevant language, who was highly proficient in English. A second native speaker, for each language, back-translated the questionnaire in English. Any difference between the two translated version of the questionnaire, in each language, were discussed among the research team and the translators, and solved through general consensus. This procedure increased the likelihood that the translations were accurate and culturally appropriate.

The questionnaire aimed to assess (i) participants’ tendency to attribute human traits to monkeys and other animals, and (ii) other variables that might influence anthropomorphic tendencies (i.e. participants’ exposure to and experience with animals, social integration, allocentric/idiocentric tendencies and other personal and demographic characteristics; for the complete questionnaire including all the questions, see supplemental information). We especially focused on monkeys, because primates are the taxon that is morphologically and behaviourally most similar and phylogenetically closest to humans, and because monkeys also naturally occurred in several areas in which we recruited participants. First, we assessed participants’ anthropomorphic tendencies with 18 questions that participants had to rate on a 5-point Likert scale (from 1=strongly disagree to 5=strongly agree). The first 10 questions corresponded to the 5 anthropomorphic items on animals from the Individual Differences in Anthropomorphism Questionnaire (IDAQ) by Waytz and colleagues,^21^ which we administered twice (i.e. once to assess participants’ tendency to attribute human traits to monkeys and once to other animals). Therefore, participants were asked to separately assess whether monkeys and other animals (1-2) have free will, (3-4) intentions, (5-6) consciousness and (7-8) minds of their own, and (9-10) experience emotions. The last 8 questions were added to further explore participants’ tendency to attribute other human traits to monkeys, and in particular whether participants considered monkeys to (11) be physically similar to humans, (12) experience pain, (13) understand others’ intentions, (14) be able to deceive others, (15) distinguish good and evil, (16) experience primary emotions like fear, happiness, anger or curiosity, (17) experience secondary emotions like shame, guilt, pride or embarrassment, and (18) be able to understand and share others’ feelings.

We assessed exposure to monkeys by asking participants about the frequency with which they encountered monkeys in the area they lived. We assessed participants’ “urban” experience with animals using 7 binary questions measuring different aspects of human-animal interactions, with a special focus on those that are more likely to occur in “urban” contexts (during safe interactions with captive, domesticated and/or virtual animals in urban contexts.^16^ For each participant, we calculated the average number of answers reporting “urban” experience with animals, obtaining a score between 0 and 1 (where 0 meant scant and 1 frequent “urban” experience with animals). We further assessed participants’ social integration with 9 questions measuring the average size of their perceived social networks. For each participant, we obtained a social integration score by summing the scores obtained for all 9 questions, which ranged from 0 to 1 (as each participant’s answer was divided by the highest number provided to that question across participants, so that a low number meant low and a high number meant high social integration, respectively). We assessed participants’ allocentric and idiocentric tendencies by using a 16-item validated questionnaire,^87^^,^^88^ which participants rated on a 5-point Likert scale. For each participant, we calculated an allocentric/idiocentric score ranging from 0 to 1, by summing the scores obtained for each question (after reversing questions measuring allocentrism) and dividing the total for the maximum score that could be obtained (so that 0 meant highly allocentric and 1 highly idiocentric participants). Finally, we collected socio- demographic information on participants’ age, gender, formal education (i.e. 1: participants that did not conclude primary school, 2: those that concluded primary school, 3: those that concluded secondary school, 4: those that had a bachelor degree, and 5: those that had higher formal degrees), religion (i.e. atheists/agnostics, Buddhists/Hindus, Christians, Muslims and other), and income (which we relevelled within countries to vary between 0 and 1, by dividing each participant’s score for the highest value provided by participants of that country, to account for variation across countries in the average income).

### Quantification and statistical analysis

We prepared two datasets for our models, the first one for questions 1 to 10 (which referred to both monkeys and other animals), and the second one for questions 11 to 18 (which only referred to monkeys). In the first dataset, we entered two lines for each study participant, one for questions on monkeys, and one for questions on other animals. In each line, we specified the country in which the data were collected, participants’ identity number, age, gender, formal education, religion, income, exposure to monkeys, experience with animals, allocentric/idiocentric tendencies and social integration. In each line, we further specified how likely (from 0 to 1) participants were to attribute free will, intentions, consciousness, minds and emotions to monkeys or other animals. In the second dataset, we entered eight lines for each study participant, one for each question about monkey attributes (i.e. physical similarity, experience of pain, understanding of others’ intentions, ability to deceive others, ability to distinguish good and evil, experience of primary emotions, experience of secondary emotions, understanding and sharing of others’ feelings; questions vi-xiii in the questionnaire). As for the first dataset, in each line we specified the country in which the data was collected, participants’ identity number, age, gender, formal education, religion, income, exposure to monkeys, experience with animals, allocentric/idiocentric tendencies, social integration, and their response (from 0 to 1) to each question. After removing lines with missing information, we ended with N=902 lines in the first dataset, and N=3643 in the second dataset.

We fitted 6 models in R (version 4.2.2),^89^ using the glmmTMB package (version 1.0.0),^90^ with a beta distribution. The first five models were based on the first dataset and included, as response, the likelihood of attributing free will (Model 1), intentions (Model 2), consciousness (Model 3), minds (Model 4) and emotions (Model 5) to monkeys or other animals. We decided to run one separate model for each of the 5 anthropomorphic items included in the IDAQ scale,^21^ rather than combining together the items into a single model, because these items capture slightly different aspects of anthropomorphism, and our test predictors might affect them in a different way (e.g. exposure to monkeys or experience to animals might affect how likely people attribute some items, but not others). In all these models, we included, as test predictors, the six 2-ways interactions between taxa (i.e. monkeys vs other animals) and participant’s education, religion, exposure to monkeys, experience with animals, social integration and allocentric/idiocentric tendencies, and their main terms. As controls, we included participants’ age, gender and income, and as random effects participants’ identity nested in country identity. The last model (Model 6) was based on the second dataset and included as response the likelihood of attributing anthropomorphic traits to monkeys. As test predictors, we included the six 2-ways interactions between anthropomorphic trait (i.e. physical similarity, experience of pain, understanding of others’ intentions, ability to deceive others, ability to distinguish good and evil, experience of primary emotions, experience of secondary emotions, understanding and sharing of others’ feelings) and participant’s education, religion, exposure to monkeys, experience with animals, social integration and allocentric/idiocentric tendencies, and their main terms. As controls, we included participants’ age, gender and income, and, as random effects, participants’ identity nested in country identity (as each participant could only be assigned to a country). In all our models, country was included as a random effect and not as predictor, because we did not have specific predictions on possible differences between countries, but we wanted to control for the effect of such differences on our test variables. Therefore, we did not expect variation across countries, but as we conducted repeated observations within each country, we included country identity as random factor.

In all models, we transformed the response variable to avoid values being exactly zero or one,^91^ and we *z*-transformed continuous variables to facilitate model convergence and interpretation of the estimates. Full models were always compared to null models (i.e., being identical to the full models, but only including controls and random factors) using likelihood ratio tests.^92^ If the full and null models differed, we used the drop1 function to assess which test predictors were significant. When interactions were not significant, we re-run the full model after removing all the non-significant interactions. In case of significant categorical predictors with more than two categories, we then used the emmeans package to run post-hoc comparisons with Tukey adjustments for multiple comparisons.^93^ Below, we only report significant post-hoc comparisons, but in supplemental information we report all the post-hoc comparisons, together with the estimates, standard errors, confidence intervals, likelihood ratio tests, degrees of freedom and *p* values for each test predictors of each full (Tables S2 and S3). Finally, we used the “DHARMa” package^94^ and the “performance” package^95^ to check model assumptions. We found no overdispersion or multicollinearity issues in any of the models presented (maximum variance inflation factors across models lacking interactions = 2.12).^96^

## Supplemental information

Document S1. Tables S1–S9

Document S2. Datasets and R script used in this study Script: R script to analyze the data with a modeling approach. Data S1: Raw experimental data for Models 1 to 5. Data S2: Raw experimental data for Model 6.
