# Supplementary material for: Experience with animals, religion, and social integration predict anthropomorphism across five countries
Source: iScience. 2025 Jun 18;28(7):112693. doi: 10.1016/j.isci.2025.112693 (PMC12432846; doi:10.1016/j.isci.2025.112693)
Supplement: Document S1. Tables S1–S9 [file mmc1.pdf]

iScience, Volume *28*

Supplemental information

Experience with animals, religion, and social integration predict anthropomorphism across five countries

Federica Amici, Jose Luis Gomez-Melara, Bonaventura Majolo, Rufino Acosta-Naranjo, Patrícia Izar, Risma Illa Maulany, Putu Oka Ngakan, Shahrul Anuar Mohd Sah, and Katja Liebal

Table S1. Information about the Spanish sample tested in two modalities. For each variable, mean ± SD and results of the Mann-Whitney exact test comparing participants' anthropomorphism, exposure to and experience with animals, social integration and allocentric/idiocentric tendencies in the Spanish sample, depending on the modality used to recruit them (i.e. in person versus online).

**Variables**	**Mean±SD**		**Mann-Whitney tests**	
	In person	Online	W	*p*-value
Anthropomorphism: free will	0.65±0.21	0.57±0.23	683	0.179
Anthropomorphism: intentions	0.74±0.18	0.71±0.21	814	0.792
Anthropomorphism: consciousness	0.61±0.24	0.71±0.21	1043.5	0.092
Anthropomorphism: minds	0.74±0.2=	0.76±0.18	893	0.682
Anthropomorphism: emotions	0.87±0.14	0.87±0.14	841.5	0.987
Exposure	1.06±0.24	1.07±0.25	763	1.000
Experience with animals	0.8=±0.18	0.8=±0.17	810.5	0.835
Social integration	1.59±0.8=	1.43±0.55	761	0.595
Allocentric/idiocentric tendencies	0.51±0.09	0.55±0.08	976	0.256

Table S2. Results for the statistical model for the probability of attributing free will (Model 1). We report estimates, standard errors (SE), confidence intervals (CIs), likelihood ratio tests (LRT), degrees of freedom (df), and *p* values for each test predictors (marked with an asterisk when significant) and for each control (in italics), with the reference category in parentheses.

**Models, test predictors & controls**	**Estimate**	**SE**	**2.5% to 97.5% CIs**	**LRT**	**df**	***p***
Intercept	3.00	0.65	1.72 to 4.28	-	-	-
Taxa (monkeys)	0.04	0.03	-0.02 to 0.11	1.89	1	0.169
Allocentrism/Idiocentrism	-0.22	0.08	-0.37 to -0.07	8.51	1	0.004*
Religion (Christians)	-0.50	0.47	-1.42 to 0.42	9.01	4	0.061
Religion (Muslims)	-0.45	0.43	-1.3 to 0.39			
Religion (Atheists/Agnostics)	-1.00	0.48	-1.94 to -0.07			
Religion (Other)	-1.24	0.56	-2.33 to -0.15			
Formal education	-0.25	0.12	-0.48 to -0.02	4.36	1	0.037*
Exposure to monkeys	0.01	0.06	-0.11 to 0.13	0.03	1	0.870
“Urban” animal experience	0.29	0.08	0.13 to 0.44	12.88	1	<0.001*
Social integration	-0.11	0.09	-0.29 to 0.06	1.64	1	0.200
Gender (male)	-0.16	0.15	-0.45 to 0.14	1.05	1	0.304
Age	-0.07	0.10	-0.27 to 0.12	0.55	1	0.457
Income	-0.06	0.08	-0.22 to 0.1	0.52	1	0.470

Table S3. Results for the statistical model for the probability of attributing intentions (Model 2). We report estimates, standard errors (SE), confidence intervals (CIs), likelihood ratio tests (LRT), degrees of freedom (df), and *p* values for each test predictors (marked with an asterisk when significant) and for each control (in italics), with the reference category in parentheses.

**Models, test predictors & controls**	**Estimate**	**SE**	**2.5% to 97.5% CIs**	**LRT**	**df**	***p***
Intercept	2.93	0.66	1.64 to 4.23	-	-	-
Taxa (monkeys)	0.06	0.03	0.01 to 0.12	4.90	1	0.027*
Allocentrism/Idiocentrism	-0.08	0.08	-0.23 to 0.07	1.03	1	0.311
Religion (Christians)	-0.46	0.47	-1.38 to 0.46	8.46	4	0.076
Religion (Muslims)	-1.05	0.45	-1.94 to -0.15			
Religion (Atheists/Agnostics)	-0.45	0.47	-1.38 to 0.47			
Religion (Other)	-0.14	0.56	-1.24 to 0.97			
Formal education	-0.22	0.12	-0.47 to 0.02	3.17	1	0.075
Exposure to monkeys	0.09	0.06	-0.03 to 0.21	1.98	1	0.159
“Urban” animal experience	0.22	0.08	0.06 to 0.38	7.15	1	0.008*
Social integration	0.02	0.09	-0.16 to 0.21	0.07	1	0.795
Gender (male)	0.04	0.16	-0.16 to 0.24	2.33	1	0.127
Age	-0.15	0.10	-0.31 to 0.01	0.18	1	0.668
Income	-0.24	0.08	-0.54 to 0.07	3.25	1	0.071

Table S4. Results for the statistical model for the probability of attributing consciousness (Model 3). We report estimates, standard errors (SE), confidence intervals (CIs), likelihood ratio tests (LRT), degrees of freedom (df), and *p* values for each test predictors (marked with an asterisk when significant) and for each control (in italics), with the reference category in parentheses.

**Models, test predictors & controls**	**Estimate**	**SE**	**2.5% to 97.5% CIs**	**LRT**	**df**	***p***
Intercept	1.91	0.56	0.81 to 3.01	-	-	-
Taxa (monkeys)*Religion (Christians)	0.25	0.26	-0.26 to 0.77			
Taxa (monkeys)*Religion (Muslims)	-0.04	0.25	-0.53 to 0.46			
Taxa (monkeys)*Religion (Atheists/Agnostics)	0.42	0.26	-0.09 to 0.93			
Taxa (monkeys)*Religion (Other)	0.34	0.32	-0.30 to 0.97			
Taxa (monkeys)	0.03	0.25	-0.45 to 0.51	23.73	4	<0.001*
Religion (Christians)	-1.07	0.40	-1.86 to -0.28			
Religion (Muslims)	-1.30	0.39	-2.07 to -0.53			
Religion (Atheists/Agnostics)	-1.04	0.40	-1.82 to -0.26			
Religion (Other)	-0.43	0.50	-1.41 to 0.54			
Allocentrism/Idiocentrism	-0.08	0.07	-0.21 to 0.06	1.30	1	0.254
Formal education	0.06	0.10	-0.13 to 0.26	0.31	1	0.580
Exposure to monkeys	0.10	0.05	-0.01 to 0.20	3.39	1	0.065
"Urban" animal experience	0.21	0.07	0.07 to 0.34	7.35	1	0.007*
Social integration	-0.15	0.07	-0.29 to 0.00	2.17	1	0.140
Gender (male)	0.10	0.14	-0.16 to 0.37	0.59	1	0.442
Age	-0.09	0.08	-0.26 to 0.07	1.25	1	0.263
Income	-0.08	0.07	-0.22 to 0.06	1.26	1	0.261

Table S5. Results for the statistical model for the probability of attributing mind (Model 4). We report estimates, standard errors (SE), confidence intervals (CIs), likelihood ratio tests (LRT), degrees of freedom (df), and *p* values for each test predictors (marked with an asterisk when significant) and for each control (in italics), with the reference category in parentheses.

**Models, test predictors & controls**	**Estimate**	**SE**	**2.5% to 97.5% CIs**	**LRT**	**df**	***p***
Intercept	2.07	0.63	0.84 to 3.29	-	-	-
Taxa (monkeys)*Religion (Christians)	0.06	0.22	-0.37 to 0.5	17.00	4	0.002*
Taxa (monkeys)*Religion (Muslims)	-0.16	0.21	-0.58 to 0.26			
Taxa (monkeys)*Religion (Atheists/Agnostics)	0.17	0.22	-0.26 to 0.6			
Taxa (monkeys)*Religion (Other)	0.03	0.28	-0.51 to 0.58			
Taxa (monkeys)	0.13	0.21	-0.28 to 0.54			
Religion (Christians)	-0.92	0.44	-1.78 to -0.05			
Religion (Muslims)	-1.18	0.43	-2.02 to -0.33			
Religion (Atheists/Agnostics)	-0.82	0.43	-1.67 to 0.03			
Religion (Other)	-0.23	0.55	-1.31 to 0.85			
Allocentrism/Idiocentrism	-0.07	0.08	-0.22 to 0.08	0.91	1	0.340
Formal education	0.12	0.11	-0.1 to 0.34	1.06	1	0.303
Exposure to monkeys	0.08	0.06	-0.04 to 0.2	1.88	1	0.170
“Urban” animal experience	0.25	0.08	0.1 to 0.4	9.89	1	0.002*
Social integration	-0.17	0.08	-0.34 to -0.01	3.56	1	0.059
Gender (male)	0.26	0.15	-0.04 to 0.56	2.84	1	0.092
Age	-0.04	0.10	-0.22 to 0.15	0.16	1	0.691
Income	-0.08	0.08	-0.23 to 0.08	0.98	1	0.323

Table S6. Results for the statistical model for the probability of attributing emotions (Model 5). We report estimates, standard errors (SE), confidence intervals (CIs), likelihood ratio tests (LRT), degrees of freedom (df), and *p* values for each test predictors (marked with an asterisk when significant) and for each control (in italics), with the reference category in parentheses.

**Models, test predictors & controls**	**Estimate**	**SE**	**2.5% to 97.5% CIs**	**LRT**	**df**	***p***
Intercept	2.77	0.61	1.58 to 3.96	-	-	-
Taxa (monkeys)	0.09	0.04	0 to 0.17	4.30	1	0.038*
Allocentrism/Idiocentrism	0.00	0.08	-0.15 to 0.14	0.00	1	0.965
Religion (Christians)	-0.49	0.42	-1.31 to 0.33	6.34	4	0.175
Religion (Muslims)	-0.83	0.41	-1.63 to -0.03			
Religion (Atheists/Agnostics)	-0.09	0.41	-0.9 to 0.71			
Religion (Other)	-0.03	0.52	-1.05 to 0.98			
Formal education	0.02	0.11	-0.2 to 0.24	0.04	1	0.842
Exposure to monkeys	0.03	0.06	-0.09 to 0.14	0.20	1	0.654
“Urban” animal experience	0.32	0.08	0.17 to 0.47	15.46	1	<0.001*
Social integration	-0.19	0.08	-0.35 to -0.02	3.84	1	0.050*
Gender (male)	-0.07	0.15	-0.36 to 0.23	0.20	1	0.651
Age	-0.02	0.09	-0.2 to 0.17	0.03	1	0.871
Income	0.03	0.08	-0.12 to 0.18	0.15	1	0.701

Table S7. Results for the statistical model for the probability of attributing anthropomorphic traits to monkeys (Model 6). We report estimates, standard errors (SE), confidence intervals (CIs), likelihood ratio tests (LRT), degrees of freedom (df), and *p* values for each test predictors (marked with an asterisk when significant) and for each control (in italics), with the reference category in parentheses.

**Models, test predictors & controls**	**Estimate**	**SE**	**2.5% to 97.5% CIs**	**LRT**	**df**	***P***
Intercept	1.06	0.42	0.23 to 1.88	-	-	-
Trait (primary emotions)*Religion (Christians)	0.32	0.38	-0.44 to 1.07	102.92	28	<0.001*
Trait (secondary emotions)*Religion (Christians)	0.67	0.38	-0.07 to 1.4			
Trait (good and evil)*Religion (Christians)	-0.10	0.37	-0.83 to 0.63			
Trait (other feelings)*Religion (Christians)	0.50	0.38	-0.24 to 1.24			
Trait (other intentions)*Religion (Christians)	0.14	0.38	-0.61 to 0.89			
Trait (pain)*Religion (Christians)	0.42	0.39	-0.34 to 1.18			
Trait (similarity)*Religion (Christians)	0.36	0.38	-0.39 to 1.11			
Trait (primary emotions)*Religion (Muslims)	0.44	0.37	-0.29 to 1.17			
Trait (secondary emotions)*Religion (Muslims)	0.86	0.37	0.15 to 1.58			
Trait (good and evil)*Religion (Muslims)	0.30	0.36	-0.41 to 1.01			
Trait (other feelings)*Religion (Muslims)	0.81	0.37	0.09 to 1.52			
Trait (other intentions)*Religion (Muslims)	0.14	0.37	-0.59 to 0.87			
Trait (pain)*Religion (Muslims)	0.66	0.38	-0.07 to 1.4			
Trait (similarity)*Religion (Muslims)	0.33	0.37	-0.4 to 1.06			
Trait (primary emotions)*Religion (Atheists/Agnostics)	0.46	0.38	-0.28 to 1.21			
Trait (secondary emotions)*Religion (Atheists/Agnostics)	0.31	0.37	-0.42 to 1.04			
Trait (good and evil)*Religion (Atheists/Agnostics)	-0.74	0.37	-1.46 to -0.02			
Trait (other feelings)*Religion (Atheists/Agnostics)	0.53	0.37	-0.2 to 1.27			
Trait (other intentions)*Religion (Atheists/Agnostics)	-0.19	0.38	-0.93 to 0.55			
Trait (pain)*Religion (Atheists/Agnostics)	0.50	0.38	-0.25 to 1.25			
Trait (similarity)*Religion (Atheists/Agnostics)	0.78	0.38	0.03 to 1.52			

Trait (primary emotions)*Religion (Other)	0.76	0.47	-0.16 to 1.68			
Trait (secondary emotions)*Religion (Other)	0.80	0.45	-0.09 to 1.69			
Trait (good and evil)*Religion (Other)	-0.06	0.46	-0.95 to 0.84			
Trait (other feelings)*Religion (Other)	1.07	0.46	0.17 to 1.96			
Trait (other intentions)*Religion (Other)	0.37	0.46	-0.53 to 1.27			
Trait (pain)*Religion (Other)	0.98	0.48	0.04 to 1.91			
Trait (similarity)*Religion (Other)	0.74	0.47	-0.18 to 1.65			
Trait (primary emotions)	0.72	0.37	-0.01 to 1.45			
Trait (secondary emotions)	-0.62	0.37	-1.34 to 0.09			
Trait (good and evil)	-0.37	0.36	-1.09 to 0.34			
Trait (other feelings)	-0.24	0.37	-0.96 to 0.48			
Trait (other intentions)	0.48	0.37	-0.25 to 1.21			
Trait (pain)	1.48	0.38	0.74 to 2.22			
Trait (similarity)	-0.04	0.37	-0.77 to 0.69			
Religion (Christians)	-0.29	0.35	-0.97 to 0.39			
Religion (Muslims)	-0.88	0.34	-1.54 to -0.22			
Religion (Atheists/Agnostics)	0.02	0.34	-0.65 to 0.69			
Religion (Other)	-0.32	0.42	-1.15 to 0.51			
Trait (primary emotions)*Exposure to monkeys	-0.20	0.05	-0.3 to -0.1	57.40	7	<0.001*
Trait (secondary emotions)*Exposure to monkeys	-0.15	0.05	-0.25 to -0.06			
Trait (good and evil)*Exposure to monkeys	-0.11	0.05	-0.2 to -0.01			
Trait (other feelings)*Exposure to monkeys	-0.20	0.05	-0.3 to -0.11			
Trait (other intentions)*Exposure to monkeys	-0.14	0.05	-0.23 to -0.04			
Trait (pain)*Exposure to monkeys	-0.35	0.05	-0.45 to -0.25			
Trait (similarity)*Exposure to monkeys	-0.08	0.05	-0.18 to 0.02			
Exposure to monkeys	0.21	0.05	0.12 to 0.31			
Trait (primary emotions)*"Urban" animal experience	0.21	0.07	0.07 to 0.34	15.14	7	0.034*
Trait (secondary emotions)*"Urban" animal experience	0.16	0.07	0.03 to 0.29			
Trait (good and evil)*"Urban" animal experience	0.05	0.07	-0.08 to 0.18			
Trait (other feelings)*"Urban" animal experience	0.15	0.07	0.02 to 0.28			
Trait (other intentions)*"Urban" animal experience	0.11	0.07	-0.02 to 0.24			
Trait (pain)*"Urban" animal experience	0.19	0.07	0.06 to 0.33			

Trait (similarity)*“Urban” animal experience	0.10	0.07	-0.03 to 0.23			
Taxa (monkeys)*“Urban” animal experience	0.21	0.07	0.07 to 0.34			
Urban” animal experience	0.06	0.06	-0.06 to 0.18			
Trait (primary emotions)*Social integration	-0.07	0.07	-0.21 to 0.07	17.33	7	0.015*
Trait (secondary emotions)*Social integration	-0.08	0.07	-0.22 to 0.05			
Trait (good and evil)*Social integration	-0.03	0.07	-0.16 to 0.11			
Trait (other feelings)*Social integration	-0.10	0.07	-0.24 to 0.04			
Trait (other intentions)*Social integration	-0.09	0.07	-0.22 to 0.05			
Trait (pain)*Social integration	-0.10	0.07	-0.24 to 0.05			
Trait (similarity)*Social integration	-0.26	0.07	-0.4 to -0.13			
Social integration	-0.01	0.07	-0.14 to 0.12			
Allocentrism/Idiocentrism	-0.07	0.04	-0.15 to 0.01	2.64	1	0.104
Formal education	0.05	0.06	-0.07 to 0.17	0.56	1	0.453
Gender (male)	0.01	0.08	-0.15 to 0.18	0.03	1	0.868
Age	-0.03	0.05	-0.13 to 0.07	0.37	1	0.543
Income	-0.01	0.04	-0.1 to 0.07	0.06	1	0.801

Table S8. Post-hoc comparisons for Models 3, 4 and 6. Results of all the post-hoc comparisons that we run (with Tukey adjustments for multiple comparisons), including the contrasts tested, the odds ratio, standard errors (SE), z ratios and *p* values for the different levels of categorical predictors (marked with an asterisk when significant).

**Models & Interaction terms**	**Contrasts**	**Odds ratio**	**SE**	***z* ratio**	***p***
**Model 3**					
Religion: Buddhists/Hindus	Other animals vs monkeys	0.97	0.24	-0.12	0.901
Religion: Christians	Other animals vs monkeys	0.75	0.07	-3.02	0.003*
Religion: Muslims	Other animals vs monkeys	1.01	0.06	0.11	0.912
Religion: Atheists/Agnostics	Other animals vs monkeys	0.64	0.05	-5.64	<0.001*
Religion: Other	Other animals vs monkeys	0.69	0.15	-1.74	0.081
**Model 4**					
Religion: Buddhists/Hindus	Other animals vs monkeys	0.88	0.18	-0.62	0.535
Religion: Christians	Other animals vs monkeys	0.82	0.06	-2.48	0.013*
Religion: Muslims	Other animals vs monkeys	1.03	0.05	0.55	0.581
Religion: Atheists/Agnostics	Other animals vs monkeys	0.74	0.05	-4.48	<0.001*
Religion: Other	Other animals vs monkeys	0.85	0.16	-0.89	0.373
**Model 6**					
Trait: deception	Buddhists/Hindus vs Christians	1.34	0.46	0.84	0.917
Trait: deception	Buddhists/Hindus vs Muslims	2.42	0.82	2.62	0.066
Trait: deception	Buddhists/Hindus vs Atheists/Agnostics	0.98	0.33	-0.07	1.000
Trait: deception	Buddhists/Hindus vs Other	1.38	0.58	0.76	0.942
Trait: deception	Christians vs Muslims	1.81	0.32	3.32	0.008*
Trait: deception	Christians vs Atheists/Agnostics	0.73	0.13	-1.81	0.368
Trait: deception	Christians vs Other	1.03	0.31	0.10	1.000
Trait: deception	Muslims vs Atheists/Agnostics	0.40	0.07	-5.05	<0.001*
Trait: deception	Muslims vs Other	0.57	0.17	-1.86	0.339
Trait: deception	Atheists/Agnostics vs Other	1.41	0.42	1.16	0.773
Trait: primary emotions	Buddhists/Hindus vs Christians	0.98	0.33	-0.08	1.000
Trait: primary emotions	Buddhists/Hindus vs Muslims	1.56	0.52	1.35	0.658
Trait: primary emotions	Buddhists/Hindus vs Atheists/Agnostics	0.62	0.21	-1.46	0.592
Trait: primary emotions	Buddhists/Hindus vs Other	0.65	0.27	-1.03	0.840
Trait: primary emotions	Christians vs Muslims	1.60	0.29	2.64	0.063
Trait: primary emotions	Christians vs Atheists/Agnostics	0.63	0.11	-2.58	0.074

Trait: primary emotions	Christians vs Other	0.66	0.21	-1.31	0.688
Trait: primary emotions	Muslims vs Atheists/Agnostics	0.39	0.07	-5.21	<0.001*
Trait: primary emotions	Muslims vs Other	0.41	0.13	-2.84	0.037*
Trait: primary emotions	Atheists/Agnostics vs Other	1.05	0.32	0.16	1.000
Trait: secondary emotions	Buddhists/Hindus vs Christians	0.69	0.23	-1.13	0.791
Trait: secondary emotions	Buddhists/Hindus vs Muslims	1.02	0.33	0.07	1.000
Trait: secondary emotions	Buddhists/Hindus vs Atheists/Agnostics	0.72	0.24	-1.01	0.851
Trait: secondary emotions	Buddhists/Hindus vs Other	0.62	0.25	-1.16	0.773
Trait: secondary emotions	Christians vs Muslims	1.49	0.26	2.29	0.147
Trait: secondary emotions	Christians vs Atheists/Agnostics	1.05	0.18	0.27	0.999
Trait: secondary emotions	Christians vs Other	0.91	0.27	-0.33	0.997
Trait: secondary emotions	Muslims vs Atheists/Agnostics	0.70	0.12	-2.05	0.241
Trait: secondary emotions	Muslims vs Other	0.61	0.18	-1.69	0.442
Trait: secondary emotions	Atheists/Agnostics vs Other	0.87	0.25	-0.50	0.987
Trait: good and evil	Buddhists/Hindus vs Christians	1.48	0.49	1.18	0.765
Trait: good and evil	Buddhists/Hindus vs Muslims	1.80	0.58	1.81	0.369
Trait: good and evil	Buddhists/Hindus vs Atheists/Agnostics	2.05	0.67	2.19	0.182
Trait: good and evil	Buddhists/Hindus vs Other	1.46	0.60	0.92	0.890
Trait: good and evil	Christians vs Muslims	1.21	0.21	1.12	0.798
Trait: good and evil	Christians vs Atheists/Agnostics	1.38	0.24	1.89	0.322
Trait: good and evil	Christians vs Other	0.99	0.30	-0.04	1.000
Trait: good and evil	Muslims vs Atheists/Agnostics	1.14	0.20	0.75	0.944
Trait: good and evil	Muslims vs Other	0.81	0.25	-0.68	0.961
Trait: good and evil	Atheists/Agnostics vs Other	0.71	0.21	-1.14	0.785
Trait: other feelings	Buddhists/Hindus vs Christians	0.81	0.27	-0.62	0.973
Trait: other feelings	Buddhists/Hindus vs Muslims	1.08	0.35	0.24	0.999
Trait: other feelings	Buddhists/Hindus vs Atheists/Agnostics	0.57	0.19	-1.68	0.446
Trait: other feelings	Buddhists/Hindus vs Other	0.48	0.20	-1.80	0.371
Trait: other feelings	Christians vs Muslims	1.33	0.23	1.65	0.469
Trait: other feelings	Christians vs Atheists/Agnostics	0.71	0.12	-2.02	0.256
Trait: other feelings	Christians vs Other	0.59	0.18	-1.79	0.379
Trait: other feelings	Muslims vs Atheists/Agnostics	0.53	0.09	-3.68	0.002*
Trait: other feelings	Muslims vs Other	0.44	0.13	-2.77	0.045*
Trait: other feelings	Atheists/Agnostics vs Other	0.83	0.24	-0.65	0.967
Trait: other intentions	Buddhists/Hindus vs Christians	1.16	0.39	0.44	0.993

Trait: other intentions	Buddhists/Hindus vs Muslims	2.11	0.70	2.26	0.159
Trait: other intentions	Buddhists/Hindus vs Atheists/Agnostics	1.19	0.39	0.51	0.986
Trait: other intentions	Buddhists/Hindus vs Other	0.95	0.40	-0.12	1.000
Trait: other intentions	Christians vs Muslims	1.82	0.32	3.40	0.006*
Trait: other intentions	Christians vs Atheists/Agnostics	1.02	0.18	0.13	1.000
Trait: other intentions	Christians vs Other	0.82	0.25	-0.65	0.967
Trait: other intentions	Muslims vs Atheists/Agnostics	0.56	0.10	-3.27	0.009*
Trait: other intentions	Muslims vs Other	0.45	0.14	-2.66	0.061
Trait: other intentions	Atheists/Agnostics vs Other	0.80	0.24	-0.75	0.946
Trait: pain	Buddhists/Hindus vs Christians	0.88	0.31	-0.37	0.996
Trait: pain	Buddhists/Hindus vs Muslims	1.25	0.42	0.66	0.965
Trait: pain	Buddhists/Hindus vs Atheists/Agnostics	0.59	0.20	-1.54	0.539
Trait: pain	Buddhists/Hindus vs Other	0.52	0.22	-1.52	0.550
Trait: pain	Christians vs Muslims	1.42	0.26	1.90	0.315
Trait: pain	Christians vs Atheists/Agnostics	0.67	0.12	-2.17	0.190
Trait: pain	Christians vs Other	0.59	0.19	-1.65	0.468
Trait: pain	Muslims vs Atheists/Agnostics	0.47	0.09	-4.09	<0.001*
Trait: pain	Muslims vs Other	0.42	0.13	-2.76	0.045*
Trait: pain	Atheists/Agnostics vs Other	0.88	0.27	-0.42	0.993
Trait: similarity	Buddhists/Hindus vs Christians	0.93	0.32	-0.21	1.000
Trait: similarity	Buddhists/Hindus vs Muslims	1.74	0.57	1.67	0.453
Trait: similarity	Buddhists/Hindus vs Atheists/Agnostics	0.45	0.15	-2.39	0.119
Trait: similarity	Buddhists/Hindus vs Other	0.66	0.28	-0.99	0.861
Trait: similarity	Christians vs Muslims	1.86	0.34	3.46	0.005*
Trait: similarity	Christians vs Atheists/Agnostics	0.48	0.08	-4.14	<0.001*
Trait: similarity	Christians vs Other	0.71	0.22	-1.12	0.798
Trait: similarity	Muslims vs Atheists/Agnostics	0.26	0.05	-7.51	<0.001*
Trait: similarity	Muslims vs Other	0.38	0.12	-3.16	0.014*
Trait: similarity	Atheists/Agnostics vs Other	1.47	0.44	1.27	0.708

Table S9. English questionnaire. List of the questions provided to all study subjects.

**ASSESSMENT OF ANTHROPOMORPHIC TENDENCIES**<br><br>Please express your degree of agreement (i.e. strongly disagree, disagree, neutral, agree, strongly agree) with the following statements:	Animals have free will
	Monkeys have free will
	Animals have intentions
	Monkeys have intentions
	Animals have consciousness
	Monkeys have consciousness
	Animals have minds of their own
	Monkeys have minds of their own
	Animals experience emotions
	Monkeys experience emotions
	Monkeys are physically similar to humans
	Monkeys experience pain
	Monkeys understand others' intentions
	Monkeys can deceive others
	Monkeys distinguish good and evil
	Monkeys experience emotions like fear, happiness, anger or curiosity
	Monkeys experience emotions like shame, guilt, pride or embarrassment
	Monkeys can understand and share others' feelings
**ASSESSMENT OF EXPOSURE TO MONKEYS**<br><br>Please indicate the frequency (i.e. never, rarely, monthly, weekly, daily) with which you encounter monkeys in your place.	
**ASSESSMENT OF "URBAN" EXPERIENCE WITH ANIMALS**<br><br>Please answer the following questions with yes or no:	Do you own animals?
	Do you consider them to be your friends?
	Do you like to spend time playing or petting them?
	Do you know movies/books with monkeys as characters?
	Did you learn anything about monkeys at school?
	Have you ever visited a zoo, a natural reserve or a similar place?
	Have you seen news about monkeys on the TV or Internet?
**ASSESSMENT OF SOCIAL INTEGRATION**<br><br>Please answer the following questions by providing a number:	How many members belong to your family?
	How many persons live in your house?
	How many people do you have, with whom you spend your leisure time?
	How many people you know, on whom you could count, if you had a problem?
	How many times do you hang out with these people, in an average week?
	How many people do you have in your Facebook?

	With how many people do you have a face-to-face conversation on a single day?
	With how many people do you have a phone/internet conversation on a single day?
**ASSESSMENT OF ALLOCENTRIC AND IDIOCENTRIC TENDENCIES**<br><br>Please express your degree of agreement (i.e. strongly disagree, disagree, neutral, agree, strongly agree) with the following statements:	I would rather depend on myself than others
	I rely on myself most of the time, I rarely rely on others
	I often do my own thing
	My personal identity, independent of others, is very important to me
	It is important for me to do my job better than the others
	Winning is everything
	Competition is the law of nature
	When another person does better than I do, I get tense and aroused
	If a co-worker gets a prize, I would feel proud
	The well-being of my coworkers is important to me
	To me, pleasure is spending time with other
	I feel good when I cooperate with others
	Parents and children must stay together as much as possible
	it is my duty to take care of my family, even when I have to sacrifice what I want
	Family members should stick together, no matter what sacrifices are required
	It is important to me that I respect the decision made by my groups
**PERSONAL AND DEMOGRAPHIC INFORMATION**<br><br>Please provide the requested information:	Gender
	Age
	Formal education (maximum grade obtained)
	Religion
	Income
